# Hearing preservation in vestibular schwannoma with retrosigmoid approach: updating retrolabyrinthine meatotomy

**DOI:** 10.1007/s00405-026-10505-4

**Published:** 2026-08-31

**Authors:** Antonio Mazzoni, Stefano Concheri, Giorgio Valentini, Elisabetta Zanoletti

**Affiliations:** 1 Section of Otorhinolaryngology, Department of Neuroscience, University of Padova, Via Giustiniani 2, Padova, 35128 Italy; 2 Formerly, Neurosurgery, University of Ferrara, Ferrara, Italy

**Keywords:** Vestibular schwannoma, Hearing preservation surgery, Retrosigmoid approach, Retrolabyrinthine meatotomy

## Abstract

**Purpose:**

Report the updated version of retrolabyrinthine meatotomy (RLM) to the whole internal auditory canal in hearing preservation surgery (HPS) via retrosigmoid approach (RS) in sporadic vestibular schwannoma (VS).

**Methods:**

Description of surgical technique of VS removal with microscopic RS approach and RLM. Fifty-six consecutive patients with intrameatal or small (up to 15 mm extrameatal size) sporadic VS, with fundus free or fundus involved by the tumor, and good hearing at diagnosis, who underwent HPS, were retrospectively reviewed. Elective (protocol) indication for HPS was ≤ 10 mm size, ≤ 30 PTA, and ≥ 70 SDS.

**Results:**

Complete VS removal was achieved in all 56 cases, with no residual tumor or recurrence. The overall hearing preservation rate was 64% (36/56 patients), and 66% (28/42 patients) in protocol cases. Hearing outcome in fundus free vs. fundus involved cases did not show significant difference. At one-year postoperative follow-up, facial nerve function was House-Brackmann (HB) grade 1 or 2 in 54/56 patients (96%) and grade 3 in 2/56 patients (4%). The rate of relevant complications was 11% (6/56), with nasal CSF leak being the most frequent; no permanent sequelae occurred.

**Conclusions:**

Microscopic RLM with three-dimensional view is a convenient option to expose the internal auditory canal up to the fundus and to remove VS irrespective of fundus involvement.

## Introduction

Hearing preservation surgery (HPS) today treats a large number of small vestibular schwannoma (VS) for which hearing preservation as well as fitness for hearing rehabilitation with cochlear implant are a reasonable goal. While an intrameatal tumor has the option of the subtemporal middle cranial fossa approach, a tumor with some extent in the cerebello-pontine angle (CPA) is more currently approached with the retrosigmoid (RS) craniotomy.

Uncovering the full internal auditory canal (IAC) without violating the labyrinth is a challenge especially in the RS approach. This condition can be addressed with the angled endoscope [1] or the microscope retrolabyrinthine meatotomy (RLM) [2–4]. The RLM involves two basic steps: an occipital craniotomy with the medial rim position matching the distal extent of the tumor in IAC [3, 4]; a surgical corridor to the full IAC obtained by drilling off of posterior-superior wall of the IAC. The fundus is commonly considered as the distal 2 mm of IAC including the lateral wall carrying the orifices of the nerves. The topic of the fundus represents an unsolved issue in terms of both surgical exposure and indication to hearing preservation via RS approach when the distal part of IAC displays tumor involvement.

The aim of the present report was to integrate our previous papers on HPS with the description of the final steps of the surgical corridor to the lateral IAC to control both the distal pole of the tumor and the portion of the facial and cochlear nerve distal to the tumor. The outcome of the procedure is summarily reported herein as related to the HPS-RLM with the rate of morbidity of the approach, completeness of tumor removal and hearing results. A further aim was to evidence how hearing preservation could be part of function focused, early surgery in vestibular neurinoma.

## Materials and methods

The RS approach with the intent of hearing preservation approach was performed by our group (senior author A.M.) in 380 cases with progressive restricting criteria in patients’ selection and surgical refinements, as detailed in previous papers [2–5].

We reported in the present series the last group of cases, operated on at the University Hospital of Padova, where we applied (i) a refinement of the surgical technique of retrosigmoid approach and (ii) strict preoperatively established inclusion criteria to candidacy to the hearing preservation approach. Fifty-six consecutive cases operated on between 2012 and 2025 were retrospectively considered.

## Surgery

**Fig. 1:**
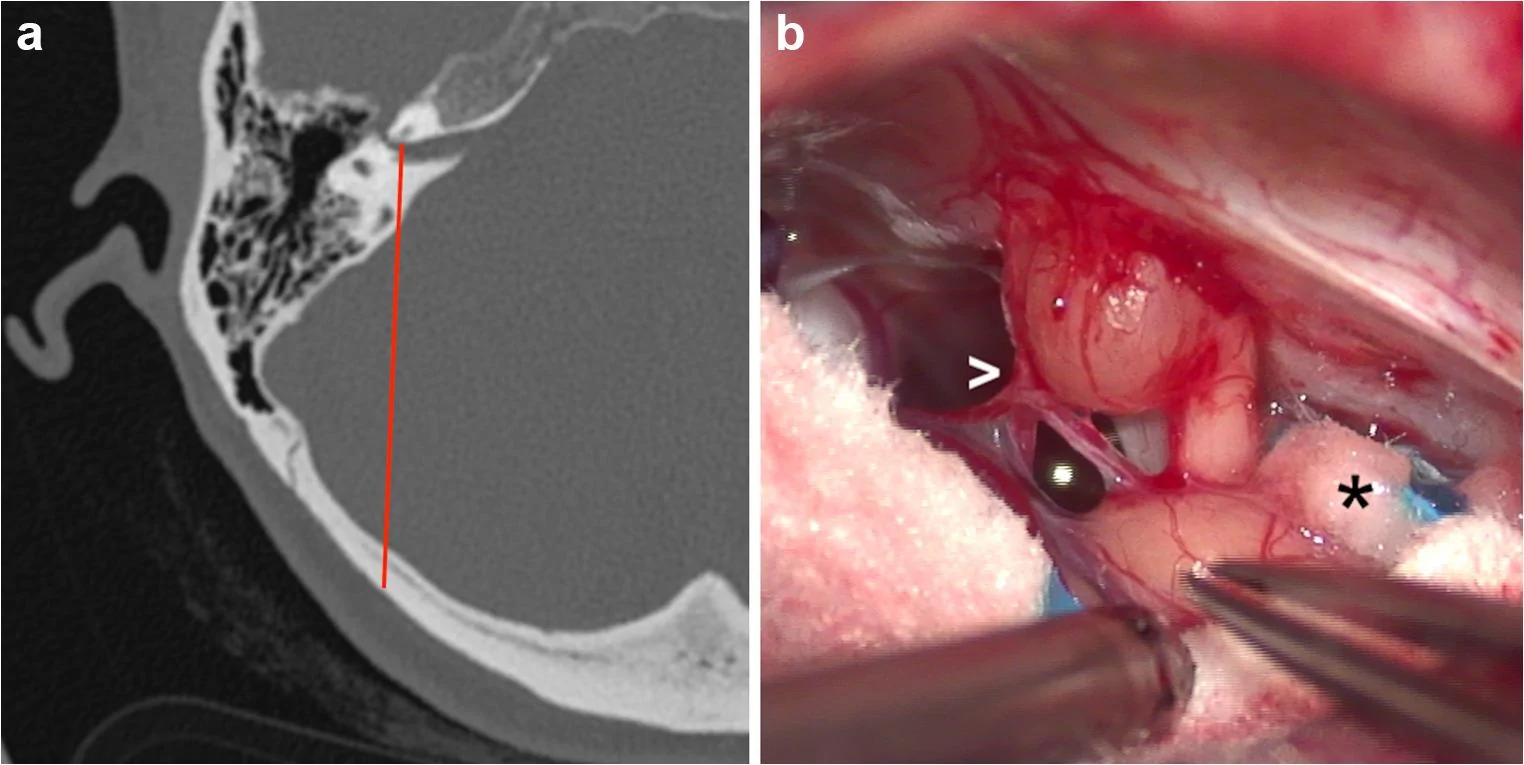
**a** The fundus-labyrinth line in right axial bone window CT. It starts at the Fallopius orifice and runs backwards tangentially to superior semicircular canal and hits the occipital squama at a spot that augmented by 10 mm indicates the posterior rim of the craniotomy. **b** CPA view with tumor, 7th and 8th nerve and AICA loop with internal auditory artery. * near field monitoring electrode; > internal auditory artery

The rationale of RLM in HPS is the correct angle of view to expose the whole IAC and bypass the unviolated labyrinth. The angle of view directs the position of the medial rim of craniotomy as well as the path to the IAC from porus to fundus by means of the retrolabyrinthine bone removal (Fig. 1a).

### Surgical anatomy

The fundus-labyrinth line, drawn on axial bone window CT scan (Fig. 1a), is the anatomical canon of RLM. It indicates the width of bone that can be removed without violating the labyrinth. It starts at the orifice of Fallopius and runs backwards tangentially to the superior semicircular canal (SCC) and hits the occipital squama at a spot, that, augmented by 11 mm, indicates the site of the medial rim of craniotomy [4] and entry of the corridor to IAC and fundus. Since fundus and labyrinth do not overlap at this angle of view, the drilling on the IAC can safely advance below the line.

In particular, the distal 1–2 mm of the superior-posterior wall of the IAC is like a ring of bone overhanging the fundus wall that can be safely removed. The final target of RLM is to expose both the distal extent of the tumor and distal portion of the cochlear nerve, as it turns anteriorly towards the modiolus. The rationale is to use the angle of view at which IAC and labyrinth do not overlap. The axial bone window CT (Fig. 1a) offers other anatomical information on obstacles and landmarks of RLM. (i) Entry of the vestibular aqueduct at the posterior wall of petrous bone and its course across bone to labyrinth [6, 7]. (ii) Architecture, cellular, compact, spongiotic, of the petrous bone in the area of drilling and presence of an obstacle like a high jugular bulb. (iii) Position and depth of semicircular canals with respect to walls of petrous bone. (iv) Shape, size of IAC and width of its roof.

### Surgical technique

**Fig. 2:**
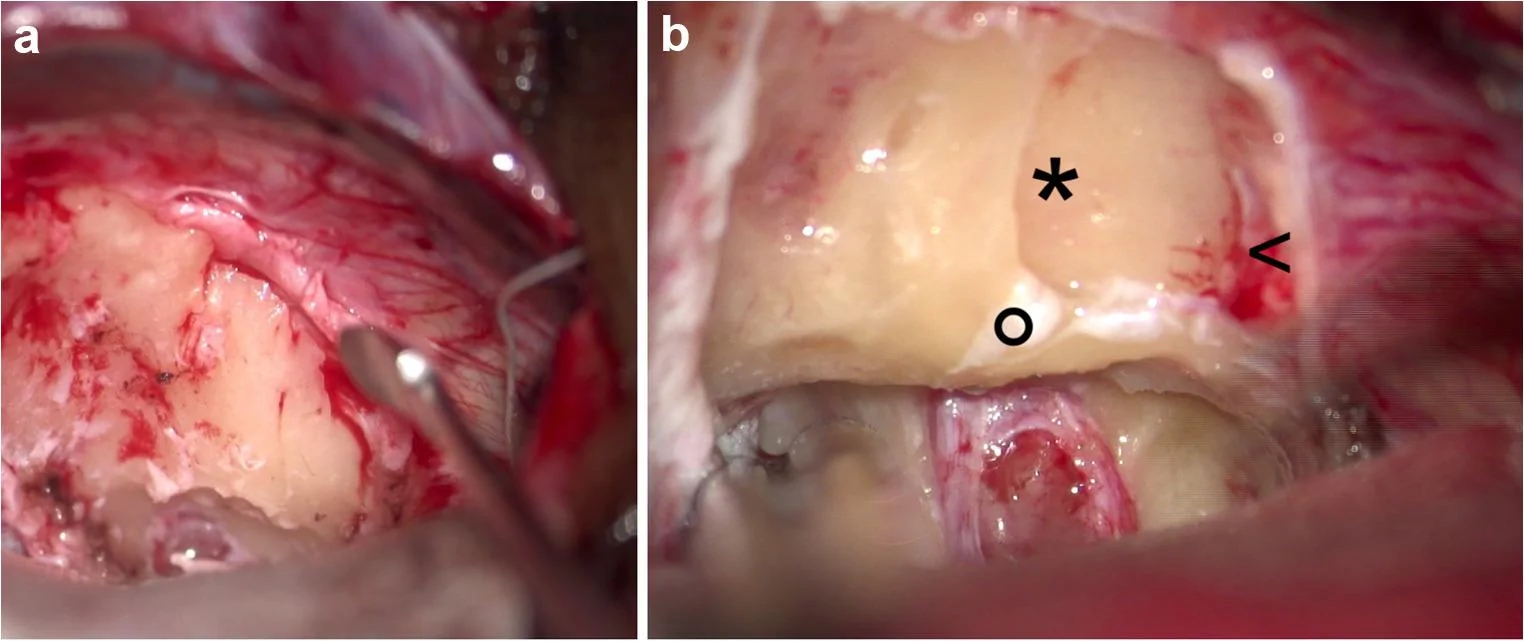
**a** Posterior side of petrous bone after removal of dura flap. Instrument points entry of endolymphatic sac and duct into slit of aqueduct. **b** Drilling on petrous bone uncovers blue line of posterior and superior semicircular canal and proximal 2/3 of internal auditory canal. * normal groove on bone, ° dura strands, < entry of vestibular aqueduct

**Fig. 3:**
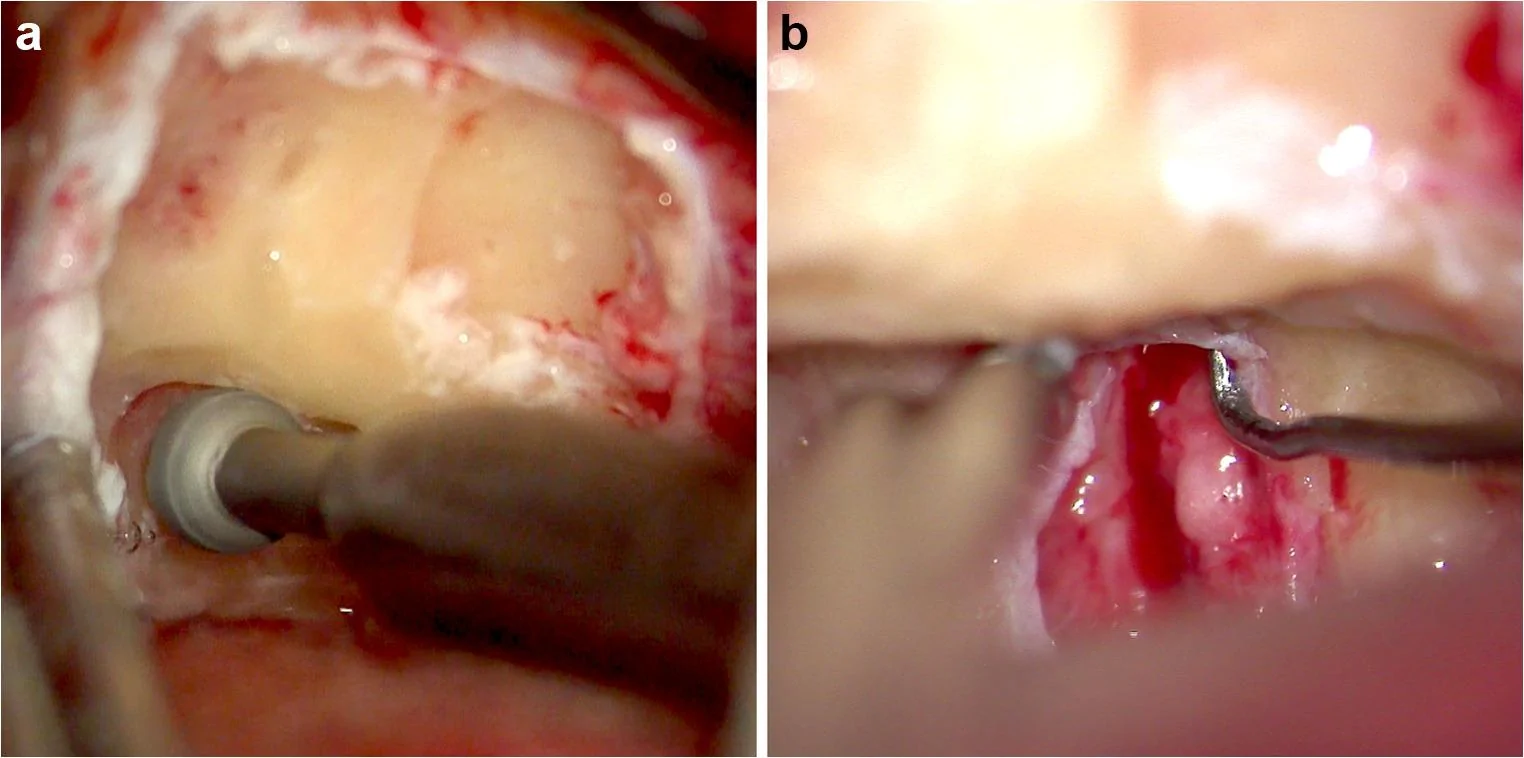
**a** Burr under the superior semicircular canal, directed to the distal roof of the IAC. **b.** IAC free of tumor. Hook checking fundus

**Fig. 4:**
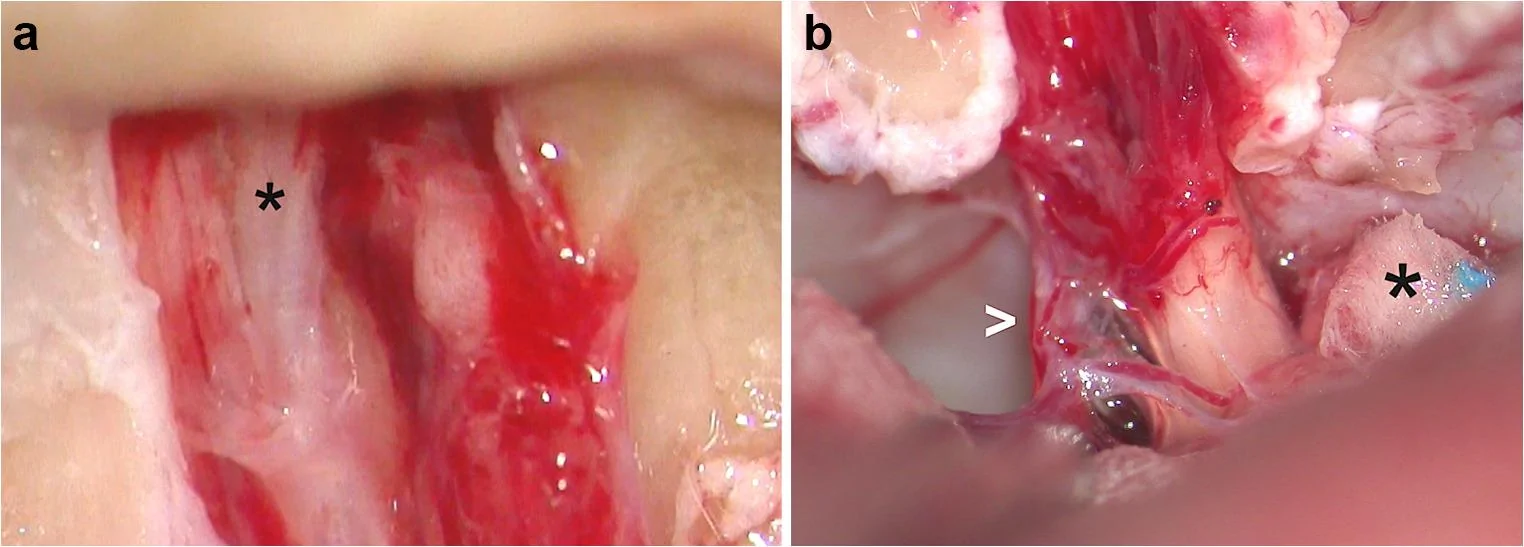
**a** Fundus exposed with 7th and 8th nerve entering their orifice. From left to right, facial nerve, transverse crista (*), cochlear nerve. **b** Final view of CPA with 7th -8th nerves, AICA and internal auditory artery. *near monitoring electrode; > internal auditory artery

The patient lies in a lateral, park-bench, position with the head turned away by 30° and flexed anteriorly. The example of a right VS of 4 mm size in CPA (Fig. 1b) is the source of the procedural pictures (Figs. 1b, 2, 3 and 4b).

Skin incision, muscles, dura and craniotomy flaps are described in previous reports [3, 4].

Handling of tumor in CPA starts at the brainstem root of 7-8th nerve and follows the tumor-nerve interface in a medial-lateral direction up to the porus and IAC. Internal auditory arteries and vein are identified and preserved as well as the arachnoid mesh connecting nerves and dura.

#### Retrolabyrinthine meatotomy

A rectangular area of dura is removed from the petrous bone to uncover the site of drilling with the following four incisions (Fig. 2b). A three quarters of circle incision around the acoustic porus, C-shaped and opened medially; two horizontal incisions, starting from the porus, the superior one is parallel to the tentorium root and the inferior one is traced from the level of the IAC floor; both horizontal incisions run laterally up to the proximity of the inner side of sigmoid sinus. The fourth, vertical incision lies just medially to the entry of the vestibular aqueduct and joins the superior with the inferior incision.

The endolymphatic sac is a flat, soft tissue organ lying within the dura at the posterior aspect of the petrous bone and enters few millimeters of bone to continue as vestibular aqueduct. It looks like a thickened dura, whiter and plicated. Its entry zone is in a bony slit 3 to 5 mm laterally to the acoustic porus. The rectangle of dura harvested from the four incisions is removed to uncover the site of drilling. The dura of the superior incision is lifted a few millimeters off the petrous ridge, carefully preserving the superior sinus. The freed dura expedites drilling of the roof of IAC and adds room to the surgical corridor to the fundus. The sequential landmarks for drilling across the petrous bone are entry of the vestibular aqueduct, bony labyrinth, posterior and superior semicircular canal, common crus, transverse crista of distal IAC. Both the CT image of the petrous bone architecture and the mental picture of the labyrinth help drilling from one landmark to the next, as follows.

- **First landmark**. Laterally lifting the dura of the vertical cut exposes the bone slit where the endolymphatic sac and vestibular aqueduct complex enter the bone (Figs. 2 and 3). The aqueduct runs medially up to the labyrinth.
- **Second landmark**. Drilling is done with a 3 mm, fine diamond burr above and away from the aqueduct, it continues across the petrous bone up to the ivory labyrinthine bone and to the blue line of the posterior semicircular canal (PSC) (Fig. 2b).
- **Third landmark**. The blue-lined PSC is followed up to the common crus of which only the superior half is blue-lined as the aqueduct crosses the lower half (Fig. 2b).
- **Fourth landmark**. Drilling medially to the PSC leads to the SSC that lies in a perpendicular plane to the PSC (Fig. 2b).
- **Fifth landmark**. The line ideally joining aqueduct-common crus-SSC marks the anterior limit of the proximal 3/4 of meatotomy. This is done by drilling the posterior and partial superior wall of IAC from the said line to porus. The dura is exposed stepwise after being lifted off its bone cover from porus to distally. With the burr under the SSC (Fig. 3a), the drilling on the posterior-superior wall can reach the transverse crista, which is seen as a bone crest on the dura surface.

- **Sixth landmark**. This final, crucial step is to remove the bone overhang that would require a blind maneuver on the vestibular quadrant in order to capture the distal tumor, as previously reported [3]. The landmark that comes in view (Fig. 4) is the distal portion of the cochlear nerve turning anteriorly to the modiolus and occasionally covered by the tumor. The orifice of the superior vestibular nerve lies tangentially out of view. Here the remainder of the IAC is a ring of bone overhanging the fundus wall, which lies in a sagittal plane. The probing with a 2 mm long, blunt, 90° degrees hook (Fig. 3b) can assess the depth of the overhang and functions as a second check for the surgeon on the amount of bone between fundus and vestibule, thus correcting potential error in setting the fundus-labyrinth line. The axial CT (Fig. 1a), like a horizontal histological section, shows the amount of bone between fundus and vestibule. As the probe may enter the vestibular nerve channel and give an excess of overhang, the drilling is to proceed for a one-millimeter step, thereon the area is checked. In practice, the fundus-labyrinth line and probing drive the last step. Drilling is to follow an axis of view running parallel to tentorium from the medial rim of craniotomy to common crus and to fundus and stop at the distal extent of the tumor where the nerves emerge off the tumor. Note that both drilling of the canal roof and lifting dura from petrous superior side create room above the fundus. If the IAC roof is thin with no additional room available, the burr can be placed in the lumen of the IAC, which is free of tumor, that was previously removed up to its distal portion.

##### Caveats

Endolymphatic sac and vestibular aqueduct represent the posterior aspect of the labyrinth and must be preserved. Their course in bone can be partially seen at CT, but is hardly visible at surgery, occasionally as a pink area on which drilling is to stop. The prudent drilling, therefore, is done superiorly to the entry of the aqueduct and up to the ivory bone of the labyrinth. Once the blue line of the superior portion of the common crus is traced, the aqueduct course is considered as lying between aqueduct entry and lower half of common crus. Lowering the residual bone, that was left as aqueduct protection, involves a risk to aqueduct and hearing. Spatial relation fundus-labyrinth is crucial for meatotomy. A 2 mm width of bone separates the vestibular quadrant of fundus from vestibule and decreases to 1 mm toward the cochlear quadrant. Drilling on fundus ring on the superior-lateral aspect prospecting the vestibular quadrant exposes the fundus, while it can endanger the labyrinth without improving control, if it extends inferiorly towards the cochlear quadrant.

##### Surgical corridor and meatotomies

It is important to outline that the course of the surgical corridor to the full IAC is obtained by drilling on the postero-superior wall and freeing of petrous dura. The trajectory of the corridor courses close to tentorium in patient’s lateral position and, with the horizontal plane, carries an acute angle posteriorly open and does not fit into a single axial CT section.

Premise that meatotomy should exceed the distal extent of the tumor at fundus and allow control of facial and cochlear nerve distally to tumor, it can be of different extent depending on the tumor. Three types of meatotomies are suggested. The *proximal* meatotomy is for a tumor extending up to the proximal half of the internal auditory canal and involves only the landmark of the orifice of vestibular and drilling on the posterior wall as much as indicated by probing between dura and bone and, if necessary, by the labyrinth landmarks of PSC and SSC. The *distal* meatotomy is for a tumor extending to the distal half of the IAC with or without fundus involvement, as assessed at T2 MRI. It may occasionally not require drilling on the distal ring if the tumor can be lifted away. In any other case, the *far distal meatotomy* allowing complete drilling of the distal ring is recommended, especially in a soft or fundus-adherent tumor.

## Patients’ selection

Criteria for HPS candidacy were progressively restricted over the years [2–5] (see Table 1) and are currently summarized in Table 2, as follows: *≤* 1 cm tumor size in the CPA, class A of AAOHNS [8] or A-B of Tokyo [9] hearing classification, normal or slightly altered auditory brain responses (ABR).

Tumor size was measured as the largest diameter in ce-MRI [9]. Pure Tone Average (PTA) included the 500-1000-2000–4000 Hz hearing thresholds. Speech discrimination score (SDS) was the maximum percentage speech recognition at speech test. Hearing classifications followed the American Academy of Otolaryngology-Head and Neck Surgery 1995 system [8] and the Tokyo 2003 system [9]. We complied with the Tokyo classification’s rule, where upgrading to the closest next better class is set for C class and lower classes (D, E) when the SDS falls in a better class than PTA. To clarify, in the Tokyo classification system class A (≤ 20dB PTA and ≥ 80% SDS) and B (≤ 30 dB PTA and ≥ 70% SDS) are excluded from the SDS upgrading rule; C class (≤ 40 dB PTA and ≥ 60% SDS) may be upgraded to class B if SDS falls in a better class than PTA, the same is for D class upgrading (≤ 60 dB PTA and ≥ 50% SDS) to C. The AAO-HNS classification system does not consider upgrading due to the SDS, and the AAHNO AB classes can be considered similar to the Tokyo A, B and upgraded C class. The Word Recognition Score (WRS) classification uses only the SDS. Gardner Robertson classification [10] measures PTA with only three frequencies (500-twice calculated 1000–2000 Hz). WRS and Gardner Robertson classification were not used.

Out of 56 cases, a group of 42 cases had size and hearing falling into our aforementioned inclusion criteria for HPS (see also Table 2). This was considered the *in-protocol* group for HPS. The remaining 14 cases slightly differed from the established inclusion criteria, as for hearing status and size, or/and altered ABR but with still detectable 5th wave; though not ideal candidates for HPS, they were considered in the “off-protocol” group and operated on with HPS procedure following strong patient’s willing and targeted counselling. Preoperative selection was not based on tumor extension within the internal auditory canal (IAC) or on fundus involvement. Fundus involvement was retrospectively evaluated on high-resolution T2-weighted MRI images using predefined criteria: the fundus was considered involved in the absence of detectable cerebrospinal fluid (CSF) and free of tumor when more than a sub millimetric CSF space was present. Retrospective assessments were performed by the authors and an in-house radiologist. A chi-square test was used to analyze hearing outcomes in respect to fundus involvement.

Surgery involved the RS approach with RLM in all the cases. Intraoperative monitoring of continuous facial nerve electromyography, Cochlear Nerve Compound Action Potentials and Auditory Brainstem Response were routine. Postoperative follow-up MRI was scheduled at 1-3-5-10y after surgery to detect recurrent or residual tumor.

Follow up ranged from 6 to 162 months. Forty-nine cases (88%) and forty-two cases (75%) cases had > 18 months and 36 months MRI follow up respectively, with no case lost. Six months at least post-operative hearing test was considered to determine HPS hearing outcome. Facial nerve outcome was set at one year. The six months evaluation was considered adequate for those cases with postoperative normal facial nerve function.

Complications were accounted in major or minor according to our classification [11] and in grades according to the Clavien-Dindo Classification [12].

 Ninety-five percent confidence intervals (95% CIs) for proportions were calculated using the exact binomial (Clopper–Pearson) method.

All study procedures complied with the principles stated in the Declaration of Helsinki “Ethical Principles for Medical Research Involving ‘Human Subjects’” and with the in-house principles. Data were retrospectively collected, grouped, and examined in agreement with Italian privacy and data laws. This study was approved by the Ethics Committee (appr. number 476n/AO/24). Informed consent was obtained for the procedure and for the study.

**Table 1:** Evolution of (a) RLM technique (landmarks of labyrinth and internal auditory canal) and (b) eligibility criteria for HPS (size and hearing) over years

*Section a*	**Period**	1976-88	1989-92	1993-97	1998-2025
	**Landmarks of RLM**	VA	VA, PSC, SSC	VA, PSC, SSC, roof of IAC	VA, PSC, SSC, roof of IAC, craniotomy
*Section b*	**Years**	1976-97	1998-2002	2002-07	2008-2025
	Size criteria*	≤30 mm	≤15 mm	≤10 mm	≤10 mm
	**Hearing criteria**	≤50dB PTA ≥50% SDS	≤30dB PTA ≥70% SDS	≤30dB PTA ≥70% SDS	≤30dB PTA ≥70% SDS ≥slightly altered ABR
	**Cases**	150	108	45	69

*VA* vestibular aqueduct, *PSC* posterior semicircular canal, *SSC* superior semicircular canal

*mm in CPA measured according to Tokyo consensus meeting

**Table 2:** Current management protocol for small vestibular schwannoma

**Tumor**	**Factors**	**Management **(HPS, TL, Obs, ±CI)
≤ 10mm in CPA	Hearing fit to HPS*	Early HPS → CI in failed HPS
	Hearing unfit to HPS	
	Assessed growth	Early TL ± CI
	No growth	Obs (MRI after 1 year)
	≥ 9mm in CPA	Obs (MRI after 6 months) or upfront TL
	Surgical risk (ageing/age, co-morbidities)	Obs or RT
	Patient’s choice	Obs or RT

* ≤30dB PTA, ≥70% SDS and normal ABR or with minor changes

*CI* cochlear implant, *CPA* cerebellopontine angle, *HPS* hearing preservation surgery, *Obs* observation, *TL* surgery via translabyrinthine approach, *RT* radiotherapy

## Results

**Fig. 5:**
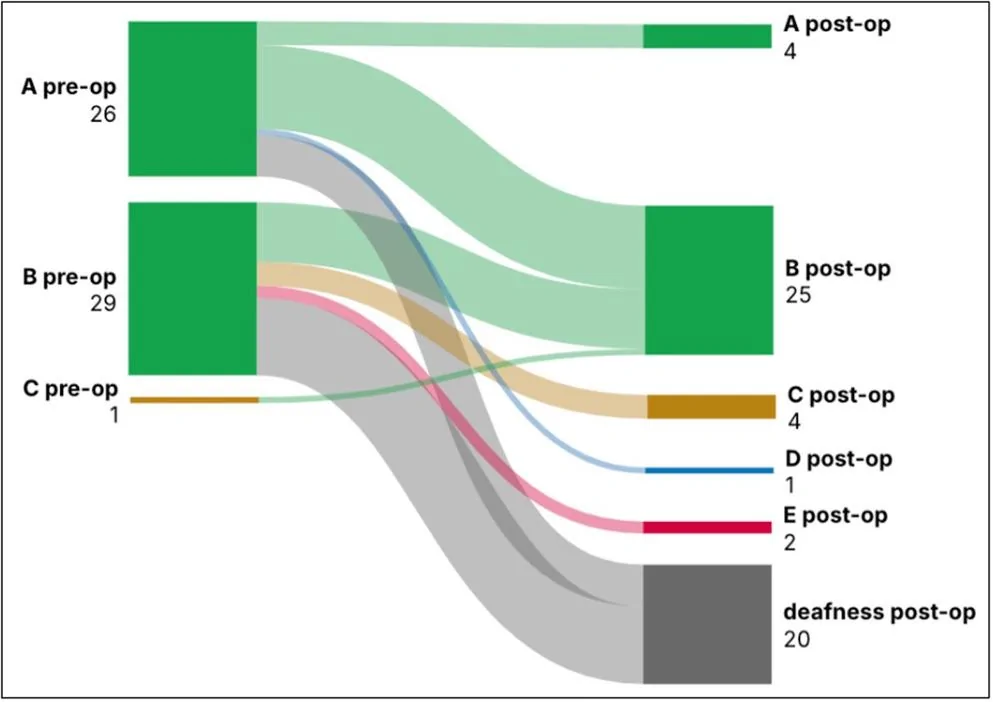
Alluvial plot of preoperative (left) and postoperative (right) hearing class of the population (*N* = 56), according to the Tokyo classification

There was no residual/recurrent tumor at MRI follow-up. The overall hearing preservation rate was 64.3% (36/56; 95%CI: 50.4–76.6%); in the in-protocol cases, it was 66.6% (28/42; 95%CI: 50.5–80.4%), as detailed in Table 3, section a. In off-protocol it was 57.1% (8/14; 95%CI: 28.9–82.3%). In the group of preserved hearing of the in-protocol cases (*n* = 28), good-serviceable hearing class A-B-C Tokyo (including upgraded classes) and A-B AAOHNS was observed in 96.4% (27/28; 95%CI: 81.7–99.9%) and 92.9% (26/28; 95% CI: 75.9–98.9%) of cases respectively. Almost no intermediate case was observed between good-serviceable hearing and deafness. An overall mismatch between SDS and PTA was observed in 37.5% (21/56; 95% CI: 25.2–51.2%) of cases, where 80%-100% SDS was to set a better level hearing class than PTA (upgraded class), following the rules of the Tokyo classification. The opposite mismatch with better PTA than SDS did not occur. Change of hearing was almost a constant outcome, in particular, slight loss in good hearing outcome and major change like deafness or severe loss in failures occurred with similar rate. In exceptionally few cases, hearing was unchanged in two and improved in one case (see Fig. 5).

Facial nerve outcome was HB 1–2 in 96.4% (54/56; 95% CI: 87.5–99.9%) of cases and HB 3 in 3.6% (2/56; 95% CI: 0.4–12.3%). Retrospective reassessment of fundus involvement was available at high resolution cT2 MRI images in all cases. Fundus was involved in 46.4% (26/56; 95% CI: 33.0–60.3%) of all cases and in 52.4% (22/42; 95% CI: 36.4–68.0%) of the in-protocol cases (see Table 3, section b). Rate of hearing preservation in fundus free vs. fundus involved cases was similar (*p* > 0.05), as detailed in Table 3. The was no case of disagreement between raters in fundus evaluation.

Complications occurred in 3, 5 and 10 patients, respectively of grade 1, 2 and 3b of the Clavien-Dindo classification [12]. They were: one case of transient cerebellar edema, occurred in the 2nd postoperative day and resolved at the 5th postoperative day after temporary CSF drainage; 5 cases of postoperative nose CFS leak, of which 2 occurred in highly pneumatized bones which were treated with surgical further waxing of temporal bone air cells, and 3 conservatively with temporary (4 days) lumbar drain. The remaining complications were transient wound swelling, all treated conservatively but in one case revised with surgery, and wound dehiscence. All these complications recovered quickly in the post-operative setting with no sequalae: after discharge, every patient went back to everyday life. Late postoperative muscular-tensive headache occurred after 3–4 months in half the cases, gradually recovered within one to two years with neck muscular exercise and training of the neck-shoulder area.

**Table 3:** Overall hearing outcome (*section a*) and in respect to fundus involvement (*section b*) in-protocol cases (*n = 42)*

	**Hearing classification**	**Class**	**n postoperative /n preoperative **	**% postoperative ** **(95% CI)**
*Section a* Overall hearing outcomes	AAO-HNS	A	12/42	28.6% (15.7–44.6%)
		A + B	26/42	61.9% (45.6–76.4%)
	Tokyo	A	4/23	9.5% (2.7–22.6%)
		A + B	12/42	28.6% (15.7–44.6%)
		A + B + B*	24/42	57.1% (40.8–72.3%)
		A + B + B* + C’	27/42	64.3% (48.0–78.4%)
			**Free fundus**	**Involved fundus**
*Section b* Hearing outcome in respect to fundus involvement	AAO-HNS (p=0.864)	A	9	5
		B	8	10
		C	1	1
		D	1	1
		deafness	11	9
	Tokyo (p=0.854)	A	3	1
		B	12	13
		C	2	2
		D	1	0
		E	1	1
		deafness	11	9

AAO-HNS: American Academy of Otolaryngology-Head and Neck Surgery; Tokyo: Hearing classification according to *New and modified reporting systems from the consensus meeting on systems for reporting results in vestibular schwannoma*, Tokyo, 2001 [9]

* 12 C class cases upgraded to B due to SDS 100% in 11 cases and 80% in 1 case

' 3 D class cases upgraded to C due to SDS 100% in 2 cases and 70% in 1 case

## Discussion

The literature on HPS is rich with thousands of articles. Despite an inherent, unavoidable heterogeneity, clinically relevant issues such as indications, results and surgical technique can be retrieved. As for preoperative indications to HPS, they gradually switched from any-size tumor to small tumor of ≤ 10 mm size in CPA, with good-fair hearing ranging between 30dB PTA/70%SDS (good hearing), and 50dB PTA/50%SDS (serviceable hearing). Conversely, attempts at hearing preservation in large tumors or any-size tumors was supported by few authors [13].

A wide range of postoperative hearing outcome was reported, as expressed by combining PTA and SDS to define the class according to the AAOHNS [8] and Tokyo system [9]. In Preet’s review [14], serviceable hearing was reported within a range from 12 to 79%, which outlined the heterogeneity of postoperative hearing outcome across studies. Better rates occurred with the preoperative class of ≤ 30dB PTA and > 70%SDS [15].

Surgical technique was rarely detailed in crucial steps and discussed in relation to outcome. When described, tumor dissection from the 7th -8th nerve was usually started at the nerve roots on the brainstem and proceeded with medio-lateral direction both in CPA and IAC. The obstacle of the labyrinth masking the lateral half of the IAC was bypassed with either an angled endoscope or a not better specified drilling of the IAC wall. Monitoring of auditory potentials of brain stem and cochlear nerve was reported and deemed necessary to prevent nerve loss.

Steps of tumor removal via retrosigmoid approach were illustrated in two representative papers. In Palva et al. [16], even today valid steps are found. Tumor removal was done in a medial to lateral direction from the CPA root of the 7th -8th nerves, but for the distal end of IAC, where dissection changed to centripetal direction. Preservation of the internal auditory arteries running anterior and inferior to 7th -8th nerves in CPA as well as along the cochlear nerve in IAC was recommended. Control of the proximal half of IAC was said to be easy, while it required shifting the microscope to the convenient angle of view for the lateral end of canal, as allowed by the medial rim of craniotomy and cerebellum. Drilling to the fundus was reported with no clear landmarks and depending mainly on the surgeon’s experience. Arachnoid strands from dura to nerves were also to be preserved, as well as the connection between cochlear and facial nerve. Laine et al. [17], from the same Institution, published a study on the anatomy of the IAC, by surgical microdissection using a special temporal bone holder and a goniometric device to simulate the angle of view of real retrosigmoid approach. Descriptive anatomy to control the fundus was reported. The present surgical technique of RLM, although based on bone window CT and histological sections of temporal bone, includes the principles reported by Laine.

The second paper was authored by Gharabaghi et al. [18]: tumor mobilization started from IAC and proceeded to CPA, where direction and time of mass reduction and dissection were modulated in order to prevent insult to nerves and let them recover. Dissection at porus was done at the end of procedure, as here stretching was more detrimental to function. The last 1–2 mm of distal IAC were left intact and controlled with an angled endoscope. In Quesnel [19], Han [20] and Bozhkov [21], a trend to better hearing preservation in fundus-free cases was observed, while it was the opposite in Nguyen [22].

Further numerous contributions did not provide a substantial change of the picture. Progress of HPS appeared to rely more on improvement of the surgical technique [23] than on better knowledge of the scarcely known pathophysiological mechanisms occurring in the inner ear during and after surgery.

The present authors’ 50-year experience with HPS involved changes in both indications, such as tumor size and hearing status, and surgical technique. This experience suggested that smaller tumor size and good preoperative hearing allowed satisfactory outcome rates to be achieved. It took 30 years to obtain surgical control of the entire IAC. In RLM, the entry of the vestibular aqueduct was the initial and only landmark leading to the internal auditory canal. Subsequent changes included the use of the PSC and SSC as landmarks [23]. The third and fourth landmarks were the roof of the IAC at the fundus and a case-by-case tailored craniotomy [4]. Table 1 shows the evolution of both the RLM technique and eligibility for HPS. The consistent dissection technique was to strictly follow the tumor surface away from the surrounding nerves, whether vestibular, cochlear, or facial.

In the present series, the overall hearing preservation rate was 64% (36/56 patients), 66% (28/42 patients) in in-protocol cases, and 57% (8/14 patients) in off-protocol cases. Facial nerve function was HB I–II in 96.4% of cases; both outcomes mirrored the previous late outcomes [3, 23]. Owing to the small number of off-protocol cases, comparison between outcomes in in- and off-protocol cases was not feasible in the present case series, although it had been performed previously [3, 4, 23]. Intraoperative monitoring, conversely, was less reliable and likely influenced the decline in hearing outcome compared with previous series [3, 4, 23]. Postoperative CSF leak was still present and pointed to surgeon-related causes, since it resolved with revised waxing or lumbar drainage. The parameters of hard/soft and adherent/loose tumor as causes of outcome were not considered because of the lack of objective measures and the presence of other concomitant factors impacting outcome.

Over recent decades, while diagnosis offered an increasing number of small tumors, the long-term outcome of the non-surgical therapies became available for reappraising the indications to therapy [24–26]. Observation and RT showed loss of hearing and facial nerve due to both the presence and growth of the tumor, and effects of radiotherapy, as well as due to a worse outcome of surgery on bigger tumors, when observation fails [11, 27], and to morbidity of secondary surgery in radiation failures [28, 29]. This promoted a new vision in which surgery took priority over non-surgical therapies, that is early TL surgery when hearing preservation was not the goal [11, 30] and HPS when hearing preservation seemed a realistic option. The principle was that a small, benign tumor postulated a therapy with definitive cure, close to zero morbidity, a quick return to normal life and hearing preservation/rehabilitation [31]. In our treatment protocol (Table 2), early surgery was being explored and HPS played an important role [11].

Fundus involvement and exposure of the whole IAC were paramount in HPS. Surgical exposure of the full auditory canal with the complete RLM was accomplished only in the last recent series since 2012. In our past experience [2, 3], there was a trend to worse hearing outcome in fundus involved cases. In the present experience, rate of hearing preservation in fundus free and fundus involved group was similar. Fundus involvement by tumor amounted to 46%, with a similar rate reported in a previous published series of 100 cases [3]. Of note, the rate of hearing outcome in those surgical series which exclude fundus-involved cases from HPS, involves a persistent bias of selection which may confound the results.

Residual tumor, absent in our series, as well as iatrogenic insult to hearing may depend on a defective control of fundus or blind dissection and difficult handling of the tumor-nerve interface. The use of an endoscope enhances visualization, but it may not perform so well in tumor handling. Other factors can influence outcome like hard tumor, fibrous adherence of tumor to nerves, thick arachnoid, tearing of the labyrinthine arteries, not to mention the cumulative load of similar insults. Comparative studies between RLM and endoscopic techniques as to fundus handling and hearing preservation were not found in the literature.

Few surgical steps crucial to fundus control were new with respect to previous reports [3, 4]. They were enlargement of distal meatotomy on the posterior and superior wall of the IAC, lifting of the petrous dura from the area of the canal roof, the probe checking with angled hook on the remaining bone to drill off at fundus as further and final step to meatotomy. Risk of insult to nerves by drilling on the internal auditory canal and especially at fundus was an obvious concern, but IAC dura lifted off its bone bed before drilling limited the exposure of nerves. Final inspection at the fundus showed that the lumen of both facial and cochlear nerve channel was larger than the nerve, thus preventing contact with the burr. Preoperative CT imaging allowed the craniotomy to be planned correctly [4]. When the tumor was found to extend more distally than at imaging, the craniotomy could always be adjusted by enlarging medially a few millimeters.

The rationale of fully exposing the IAC lies in the advantages provided by a microsurgical two-hands technique, adequate surgical room for handling tumor and instruments, constant view control of the interface tumor-nerve, feasibility of HPS in the tumor fundus-involved cases. Spontaneous relaxation of cerebellum as an effect of perioperative transient CSF lumbar drainage, as adopted in the last 20 cases, further allowed to avoid retraction by instruments and provide easier visualization and drilling of the IAC walls.

CSF transient perioperative drainage represented a progress of HPS, promoting both uneventful surgery and postoperative course. Intraoperative monitoring, conversely, was less reliable in the present series and likely influenced hearing outcome with respect to our previous series [3, 4]. Artefacts confounding intraoperative measurements are a current issue and proportional with the increasing use of technology in surgery as well as in the anesthesia monitoring tools. Despite the occurrence of few complications with favorable outcome, no residual tumor, and overall good-fair hearing preservation, room for further progress in function-focused therapy remained. The hearing preservation rate was counterbalanced by the fact that loss of hearing was almost constant. Good hearing outcome cases showed a slight loss from the preoperative status in almost all but three cases, that is unchanged hearing in two and improved in one case. In failures, major changes like deafness or severe hearing loss occurred with similar rate.

Two events were worth noting in hearing outcome: few intermediate cases were observed between status of serviceable hearing and deafness, i.e. 5% of in-protocol, and 14% in off-protocol cases respectively; in all the remaining cases, it appeared that hearing was well preserved, or otherwise lost. The second event was in the mentioned mismatch between the PTA and SDS.

The RLM had, in our experience, a long course from the initial [2] up to the present technique and remained only one of the obstacles to preserving functional cochlear nerve, spiral ganglion and Corti’s organ. Caring of blood supply to labyrinth and nerves [31] may be one of the next goals in HPS.

The main weaknesses of the present study are (i) the retrospective nature, although it is the only acceptable from an ethical point of view; (ii) the twelve-year time course, involving a stable surgical approach but changes due to the progressively increased experience and expertise; (iii) the outcome unavoidably biased by the patient’s incontrollable reasons for access to authors or institution.

## Conclusions

Fundus involvement by VS is a frequent event and the RLM in the RS approach can remove the tumor with a similar outcome as in fundus free cases. Direct, stereoscopical control of the internal auditory canal up to the fundus can be achieved with a planned individually-dependent adequate surgical corridor and angle of view. The fundus-labyrinth line, indicating the corridor from craniotomy to fundus as given by measured craniotomy on definite landmarks, provides the further sequential surgical steps to perform and complete the meatotomy. Using the microsurgical technique for the full procedure of hearing preservation surgery in retrosigmoid approach can be a convenient option.
